# Methotrexate-Loaded Gelatin and Polyvinyl Alcohol (Gel/PVA) Hydrogel as a pH-Sensitive Matrix

**DOI:** 10.3390/polym13142300

**Published:** 2021-07-14

**Authors:** Muhammad Akhlaq, Abul Kalam Azad, Inam Ullah, Asif Nawaz, Muhammad Safdar, Tanima Bhattacharya, A. B. M. Helal Uddin, Syed Atif Abbas, Allan Mathews, Sukalyan Kumar Kundu, Mireia Mallandrich Miret, H. C. Ananda Murthy, H. P. Nagaswarupa

**Affiliations:** 1 Faculty of Pharmacy, Gomal University, Dera Ismail Khan 29220, Pakistan; dr.akhlaq@gu.edu.pk (M.A.); 01drazad@gmail.com (I.U.); asifnawaz676@gmail.com (A.N.); safdarlaghari10@gmail.com (M.S.); 2 Advanced Drug Delivery Laboratory, Department of Pharmaceutical Technology, Faculty of Pharmacy, International Islamic University Malaysia (IIUM), Kuantan 25200, Pahang, Malaysia; 3 Innovation, Incubation & Industry (I-Cube) Laboratory, Techno India NJR Institute of Technology, Udaipur 313003, India; btanima1987@gmail.com; 4 Analytical and Bioanalytical Research Laboratory, Department of Pharmaceutical Chemistry, Faculty of Pharmacy, International Islamic University Malaysia (IIUM), Kuantan 25200, Pahang, Malaysia; 5 Faculty of Pharmacy, Quest International University, Ipoh 30250, Perak, Malaysia; 6 Department of Pharmacy, Jahangirnagar University, Savar, Dhaka 1342, Bangladesh; skkbd415@juniv.edu; 7 Department of Pharmacy, Pharmaceutical Technology and Physical-Chemistry, Faculty of Pharmacy and Sciences Food, University of Barcelona, 08028 Barcelona, Spain; mireia.mallandrich@ub.edu; 8 Department of Applied Chemistry, School of Applied Natural Science, Adama Science and Technology University, Adama P.O. Box 1888, Ethiopia; anandkps350@gmail.com; 9 Innovation Cell Sarojaayudh Vastram Bharath Pvt. Ltd., Bengaluru 560039, India; nswarupa@davangereuniversity.ac.in

**Keywords:** methotrexate, gelatin, polyvinyl alcohol, hydrogel, pH-responsive, targeted delivery, colon cancer

## Abstract

The aim was to formulate and evaluate Gel/PVA hydrogels as a pH-sensitive matrix to deliver methotrexate (MTX) to colon. The primed Gel/PVA hydrogels were subjected to evaluation for swelling behavior, diffusion coefficient, sol-gel characteristic and porosity using an acidic (pH 1.2) and phosphate buffer (PBS) (pH 6.8 & pH 7.4) media. Fourier transform infrared spectroscopy (FTIR) and thermal gravimetric analysis (TGA) were performed to evaluate the chemical compatibility of the Gel/PVA hydrogel. The shape alteration and release of Gel/PVA hydrogel was conducted at pH 1.2, pH 6.8 and pH 7.4. The drug release kinetic mechanism was determined using various kinetic equations. The physicochemical evaluation tests and drug release profile results were found to be significant (*p* < 0.01). However, it was dependent on the polymers’ concentration, the pH of the release media and the amount of the cross-linking agent. Hydrogels containing the maximum amount of gel showed a dynamic equilibrium of 10.09 ± 0.18 and drug release of 93.75 ± 0.13% at pH 1.2. The kinetic models showed the release of MTX from the Gel/PVA hydrogel was non-Fickian. The results confirmed that the newly formed Gel/PVA hydrogels are potential drug delivery systems for a controlled delivery of MTX to the colon.

## 1. Introduction

The increasing incidence of colorectal cancer (CRC) has started a growing concern, as it had been reported as the third most commonly diagnosed cancer in the global population worldwide [1,2]. It was also recently reported in the National Cancer Registry Report as the most commonly diagnosed cancer in Malaysian men, while being the second most commonly diagnosed cancer in Malaysian women [2]. The conventional oral cancer chemotherapy for colorectal cancer often met obstacles in delivering drugs to the colon site [3,4,5] because of the poor absorption and the degradation of the drug in the upper gastrointestinal tract (GIT) before reaching the desired target site [6,7,8]. Hence, compared to conventional cancer chemotherapy, the oral colon-targeted drug delivery system (CDDS) started to gain more advantages by providing optimal therapeutic concentrations of anti-cancer agents to the targeted sites of action with dose sparing [9,10,11].

To create a successful CDDS for an improved therapeutic effect at the site of action, a drug delivery system needs to be able to protect the anti-cancer drug from acid degradation or release in the upper GIT to achieve the targeted drug release in the colon [12,13]. Among the modern drug delivery systems, hydrogel is considered to be one of the ideal pharmaceutical dosage forms to be used as a targeted drug delivery carrier for cancer therapy [14]. The ideal properties of hydrogel have made it easy to incorporate with chemotherapy agents and enable it to reversibly shift the sol-gel status depending on the stimulus (pH and temperature) [15]. Furthermore, a pH-responsive hydrogel can also act as a smart drug delivery system to tumor sites in a controlled-release manner. Smart hydrogels show changes in swelling behavior due to variations in environmental conditions such as temperature, pH, electric field and solvent composition. Hydrogels can be defined in number of different ways [16]. 

MTX is a classical chemotherapy agent to treat several disorders such as irritable/inflammatory bowel disease, ulcerative colitis, rheumatoid arthritis [17], lymphoma and leukemia [18], head and neck cancer [19], osteogenic sarcoma [20], choriocarcinoma [21,22], psoriasis [23] and acute lymphocytic leukemia in children [24,25]. MTX is also prescribed for CRC treatment, but its oral dosage forms usually encounter obstacles in delivering drugs to the colon site due to its poor bioavailability and the degradation of the drug in the upper GIT (stomach) before reaching the desired target site. It will be metabolized in the stomach after being consumed orally and be unable to reach the targeted site in the colon to deliver the drug effectively [26]. Hence, the incorporation of MTX with controlled-release carriers as targeted drug delivery systems could help in reducing the MTX dose and maximizing the therapeutic effect with lesser side effects [15].

To improve the targeted drug delivery of MTX, Gel and PVA have been incorporated as important components of a pH-sensitive hydrogel in this study due to their unique physicochemical properties and biodegradability [27]. Gel is a protein-based natural polymer that is derived from collagen through the process of acid and alkaline hydrolysis [28]. It is widely used by manufacturers in the development of pharmaceutical and biomaterial products nowadays. The cationic and anionic functional side groups on the molecular chains of Gel are readily available to be chemically modified or cross-linked [29]; hence, these properties have been useful in developing a targeted drug delivery system [30]. In addition to that, Gel also shows changes in mechanical properties and swelling behavior in response to various stimuli, such as temperature and pH. PVA is also a hydrophilic polymer widely used as a hydrogel due to its excellent chemical stability along with good biocompatibility [31]. The mechanical properties of PVA can be adjusted via several methods, most of them rely upon thermal transition where crystallization of a PVA solution occurs through non-covalent intermolecular bonds to develop into a cross-linked polymer network structure. The formation of hydrogen bonds between Gel and PVA had been hypothesized to contribute toward the high resilience of hydrogel on the site of action because of the supramolecular network [32,33]. In the present research work, the MTX-loaded Gel/PVA hybrid hydrogel was formulated first with different polymers to cross-link the ratios to provide protection for the drug, specifically at a lower pH (stomach). The formulations were subjected to different physicochemical evaluation tests and in vitro release profiles.

## 2. Materials and Methods

### 2.1. Materials

Active methotrexate was received as a gift sample from Wilshire Pharma, Pvt limited (Lahore, Punjab 54700, Pakistan). Polyvinyl alcohol and gelatin were obtained from Fluka Biochemika (Fluka Biochemika, 9471 Buchs, Switzerland). Glutaraldehyde, hydrochloric acid, potassium phosphate monobasic and potassium chloride were purchased from Sigma Aldrich (Sigma Aldrich, Darmstadt, Germany); sodium hydroxide was supplied by Icon Chemicals (Icon Chemicals, Ludhiana, Punjab, India) and distilled water was used for the buffer preparations. All these chemicals were of analytical grade and were used without further purification. 

### 2.2. Methods

#### 2.2.1. Preparation of Gel/PVA Hybrid Hydrogel

For preparation of Gel/PVA hybrid hydrogel, two separate solutions (A and B) were prepared, using previous methods with slight modifications [34]. For preparation of solution (A), 1.5, 2 and 2.5 g of PVA were added to 20 mL of distilled water in a 50-milliliter beaker and placed on a magnetic stirrer. The polymer and solvent mixture then heated from 60 to 70°C for up to 30 min with continuous stirring at 200 rpm, until a clear solution formed. The final volume was made by adding a specific quantity of distilled water. To prepare solution (B), 9, 8, 7.5 and 7 g of Gel were added to 25 mL of distilled water in a separate 50-milliliter beaker and placed on a magnetic stirrer. The polymer and solvent mixture were then heated from 30 to 35°C for 30 min with continuous stirring at 200 rpm, until a clear solution formed. The final volume was made by adding a specific amount of distilled water. Then, the two solutions (A) and (B) were mixed in such a way that solution (B) was added drop wise into solution (A) with continuous stirring at 200 rpm for up to 30 min at 30 °C, for complete mixing of the two solutions. A total of nine (*n* = 9) formulations were prepared, glutaraldehyde was added to three of the formulations in a drop wise manner. The hybrid gel formulations were prepared with various concentrations of Gel and PVA. It was poured into a test tube and left at room temperature until completely congealed. The congealed gels were taken from the test tube, cut into 5-millimeter sized discs and dried in a Petri dish at room temperature.

#### 2.2.2. Dynamic and Equilibrium Swelling Characteristic

The weight of dried hydrogel discs was measured before they were immersed in buffer media at pH of 1.2, 6.8 and 7.4. [35]. The weight of swollen hydrogels was measured at the specific time interval of 0.5, 1, 1.5, 3, 5, 6, 7 and 8 h. The dynamic swelling of hydrogel was determined according to the following Equation (1):

(1) $$S=\frac{W_{w}}{W_{d}}$$

where “S” represents dynamic swelling, “W_d_” represents the weight of the dry disc and “W_w_” represents the weight of the wet disc.

To obtain the average dynamic swelling, the sum of the total number of dynamic swelling values was divided by the whole number of formulation sampling. The following Equation (2) was used:

(2) $$S\left(\mathrm{Avg}\right)=\frac{S1+\;S2+\ldots \ldots \ldots \ldots \ldots \ldots \ldots \ldots ..+\;S8}{\mathrm{Total}\;\mathrm{number}\;\mathrm{of}\;\mathrm{samples}}$$

where “S(Avg)” represents average dynamic swelling. 

After the completion of the dynamic swelling study, the already immersed hydrogel discs of Gel/PVA were left in the buffer medium, mostly for about 24 h, until they attained a constant/equilibrium weight. The following Equation (3) was used:

(3) $$S\left(\mathrm{Eq}\right)=\frac{W_{e}}{W_{d}}$$

where “S(Eq)” represents the equilibrium swelling, “W_e_” represents the weight of swollen discs after 24 h of swelling and “W_d_” represents the weight of the dry disc before swelling.

#### 2.2.3. Diffusion Coefficient

The diffusion coefficient represents the quantity of the solvent that diffuses through a unit area of the gel in a unit time through a concentration gradient [36]. The following parameters depend on the partial mobility of the solvent, which can be determined by the following Equation (4):

(4) $$D=\pi {\left[\frac{h\theta }{4}q\left(\mathrm{eq}\right)\right]}^{2}$$

where “D” represents the diffusion coefficient, “q(eq)” represents the equilibrium swelling of the hydrogel disc, “θ” represents the slope of the linear part of the swelling curve, while “h” represents the height (distance between the top and bottom) or thickness of the hydrogel discs.

#### 2.2.4. Sol-Gel Analysis

In this method, discs were accurately weighted and placed in tap water for 72 h. After 72 h, the individual discs were removed and kept at room temperature until they were completely dry. The discs were weighed again, the sol and gel fraction percentages were determined with the following Equations (5) and (6) [37]:

(5) $$\mathrm{Sol}\;\mathrm{fraction}\left(\%\right)=\frac{W_{1}-W_{2}}{W_{1}}\times 100$$

(6) Gel fraction(%)=100−Gel fraction

where “W_1_” represents the weight of the disc prior to immersion in the water and “W_2_” represents the weight of the disc after 72 h.

#### 2.2.5. Porosity Measurement 

To determine the porosity of Gel/PVA hydrogel discs, each disc was measured before immersion in absolute ethanol for 24 h. Each disc was then removed, and the excess ethanol wiped off with tissue paper to measure the weight. The porosity percentage was determined using the following Equation (7) [38]:

(7) $$\%\mathrm{Porosity}=\frac{M_{2}-M_{1}}{\rho V}\times 100$$

where “M_2_” represents the mass of the disc after removal from the ethanol solvent, “M_1_” represents the mass of the disc before immersion in the ethanol solvent, “ρ” represents the density of the absolute ethanol and “V” represents the volume of the hydrogel disc.

#### 2.2.6. Method of Drug Loading (In Situ Method)

In this method, the Gel/PVA hybrid hydrogel solutions were prepared in different concentrations. The drug, MTX, was added to each solution with continuous stirring on a magnetic stirrer for 40 min. Then, drug-loaded formulations were congealed, removed, cut into discs, and dried with the same procedure used for the drug-unloaded formulations.

#### 2.2.7. Drug Release

The dissolution apparatus of the paddle method was used [39]. Different buffer solutions at pH of 1.2, 6.8 and 7.4 were prepared. A buffer medium (500 mL) was added to each flask of dissolution apparatus, and a disc was immersed in each flask. The speed of the paddle rotation and the temperature was set at 100 rpm and 37 °C, respectively. At specific time intervals (0.5, 1, 2, 3, 4, 5, 6, 7, 8, 9, 10, 11 and 12 h), 5 mL of sample was withdrawn from each flask and replaced with a fresh 5 mL of the same buffer solution to maintain the sink condition. The drug release was determined using a UV–vis spectrophotometer (Shimadzu UV-1800) at a λ max of 325, *n* = 3. 

### 2.3. Characterization 

#### 2.3.1. Fourier Transform Infrared Spectroscopy (FT-IR)

FT-IR spectra were recorded from 400–4000 cm^−1^ with a resolution of 1 cm^−1^ using a PerkinElmer Spectrum 100 spectrophotometer (Perkin Elmer Corp., Norwalk, CT, USA). The spectra were processed using the SpectraGryph 1.2 spectroscopy software (Am Dummelsmoos 2887561, Oberstdorf, Germany) [40].

#### 2.3.2. Thermal Gravimetric Analysis (TGA)

Thermal gravimetric analysis of the Gel, PVA, MTX-loaded and unloaded hydrogel was conducted [41].

### 2.4. Statistical Analysis

Statistical analysis was performed using a one-way Analysis of Variance (ANOVA) and *t*-test (SPSS version 20, IBM, Armonk, NY, USA). All the formulations were calculated in triplicate (*n* = 3) and data were expressed as mean ±S.D. A *p* value *<* 0.01 was considered as a significant difference. 

## 3. Results

### 3.1. Behavior of Swelling Study 

**Figure 1:**
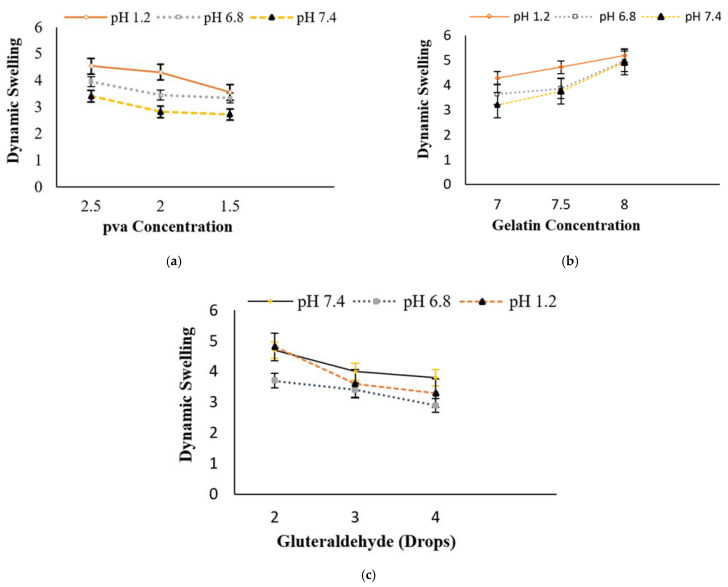
Dynamic swelling of Gel/PVA hydrogel with (**a**) decreasing concentration of PVA, (**b**) increasing concentration of Gel and (**c**) increasing quantity of glutaraldehyde. Data are expressed as mean ± S.D. and *p* < 0.01 is considered statistically significant.

In general, two major factors control drug release from swelling controlled matrix systems, including (i) the rate of aqueous medium infiltration into the matrix, followed by a relaxation process (hydration, gelation or swelling); and (ii) the rate of matrix erosion [42]. The Gel concentration was kept constant in formulations A1 to A3, while the PVA concentration varied between 1.5 g, 2 g and 2.5 g. The formulations were evaluated to determine the effect of the decreasing concentration of PVA on the swelling rate. Sample A1 showed the lowest dynamic swelling of 4.54 ± 0.23 at pH 1.2, while showing 3.41 ± 0.10 at pH 7.4 and the highest equilibrium swelling of 9.35 ± 0.35 at pH 1.2, as compared to A2 and A3. Sample A2 showed the highest equilibrium swelling of 9.10 ± 0.27 at a pH 1.2, while showing the least amount of swelling at pH 7.4. A3 depicted the minimum amount of swelling (dynamic and equilibrium) in all the pH levels, as compared to A1 and A2 with an increasing concentration of PVA and vice versa. The swelling ratio was found to decrease from A1 to A3, as shown in Figure 1a. In the case of Gel, the phenomenon was the opposite, the swelling ratio augmented as the concentration of Gel was increased. More interestingly, due to the Gel’s incorporation, the hydrogels become pH sensitive and showed a maximum amount of swelling at pH 1.2. When the PVA concentration was kept constant and the Gel concentration was increased gradually from sample A4 to sample A6, an increase was noted in the swelling profile of the Gel/PVA hydrogel samples. An increased Gel concentration in sample A6 showed a maximum amount of equilibrium swelling of 10.09 ± 0.18 and dynamic swelling of 5.20 ± 0.13, as compared to samples A5 and A4. The swelling ratio was found to decrease from A4 to A6, as shown in Figure 1b. From sample A7 to A9, the effect of the concentration of glutaraldehyde (cross-linker) was investigated by keeping the concentration of both the PVA and Gel polymers constant. Figure 1c shows that, by increasing the amount of glutaraldehyde, the swelling ratio of the Gel/PVA hydrogels decreased. Sample A9 showed an equilibrium swelling of 7.80 ± 0.49 at pH 1.2 and 3.10 ± 0.50 at pH 7.4, while sample A7 showed the maximum amount of equilibrium swelling of 7.98 ± 0.14 at pH 1.2 and 4.09 ± 0.45 at pH 7.4. The maximum amount of equilibrium swelling shown by sample A8 was 7.92 ± 0.78 at pH 1.2 and 3.92 ± 0.31 at pH 7.4. The swelling profile ratio was found to be A7 > A8 > A9, as shown in Figure 1c. Sample A6 showed the highest equilibrium swelling of 10.09 ± 0.18 and dynamic swelling of 5.20 ± 0.13 at pH 1.2 containing Gel/PVA. Sample A6 showed a significantly (*p* < 0.01) higher degree of swelling among all the formulated samples, thereby exhibiting a controlled release of MTX.

### 3.2. Sol-Gel Analysis

**Figure 2:**
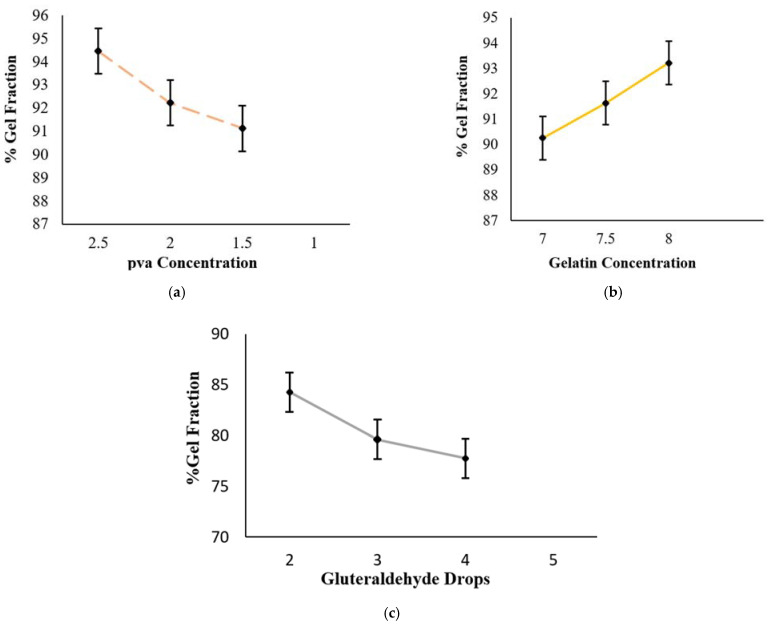
Percentage of gel fraction of Gel/PVA Hydrogel with (**a**) decreasing concentration of PVA, (**b**) increasing concentration of Gel, (**c**) increasing quantity of glutaraldehyde. Data are expressed as mean ± S.D. and *p* < 0.01 is considered statistically significant.

To determine the non-crossed linked polymer in the formulation, a sol-gel method was adopted. The sol-gel portion is directly proportional to the concentration of the polymers, i.e., PVA and Gel, and is inversely proportional to the quantity of the cross-linker. The sol-gel fraction percentage was found to be 94.46 ± 0.30% for A1, 92.22 ± 0.30% for A2, 91.13 ± 0.53% for A3, 90.25 ± 0.39% for A4, 91.63 ± 0.59% for A5, 93.21 ± 0.40% for A6, 84.27 ± 0.59% for A7, 79.61 ± 0.48% for A8 and 77.75 ± 0.12% for A9. Among all the nine formulations, the highest degree of sol-gel fraction was found for sample A1, a lower degree was found for sample A6, and the lowest degree of sol-gel fraction was found for sample A9. Figure 2a represents the decreased sol-gel fraction percentage due to lower concentrations of PVA polymers. Figure 2b shows that an increased concentration of Gel presents a maximum sol-gel fraction percentage in sample A6 (93.21 ± 0.40%) and a minimum for A4 (90.25 ± 0.39%), while Figure 2c shows that the highest concentration of the cross-linker (glutaraldehyde) results in the lowest sol-gel fraction, as can be seen in sample A9 (77.75 ± 0.12%).

### 3.3. Diffusion Coefficient

The diffusion coefficient was determined through the interaction capability of the hydrogels and solvents used over a period based on the calculation of the unit concentration gradient. In this study, the diffusion coefficient of formulation A1 was found to be 0.0148 ± 0.0061 (cm^2^/s); followed by A2, 0.0119 ± 0.0081 (cm^2^/s); A3, 0.0102 ± 0.0015 (cm^2^/s); A4, 0.0096 ± 0.0031 (cm^2^/s); A5, 0.0105 ± 0.0017 (cm^2^/s); A6, 0.0176 ± 0.0021 (cm^2^/s); A7, 0.0195 ± 0.0014 (cm^2^/s); A8, 0.0161 ± 0.0042 (cm^2^/s); and A9, 0.0107 ± 0.0037 (cm^2^/s). At a lower concentration of PVA, a decrease in the diffusion coefficient was recorded in formulations A1 to A3, while increasing the concentration of Gel led to an increasing diffusion coefficient in formulations A4 to A6. In contrast, an increasing glutaraldehyde concentration resulted in a lower diffusion coefficient, as shown in formulations A7 to A9. The diffusion coefficient might be directly proportional to the polymer concentration and inversely proportional to the concentration of the cross-linker (glutaraldehyde) [43].

### 3.4. Measurement Porosity

The effect of the different concentrations of the polymers (Gel/PVA) and of the cross-linker in the hydrogel formulations can be investigated by the porosity of various samples. All the formulations of Gel/PVA hydrogel were evaluated for porosity and experiments were performed in triplicate for the means and standard deviations. The percent (%) of porosity of sample A1 was found to be 30.76 ± 1.350, sample A2 was 24.75 ± 1.327, sample A3 was 23.32 ± 1.054, sample A4 was 20.36 ± 1.388) sample A5 was 22.63 ± 1.108, sample A6 was 28.27 ± 1.387, sample A7 was 18.35 ± 1.083, sample A8 was 16.66 ± 1.4512 and sample A9 was 10.60 ± 0.450.

**Figure 3:**
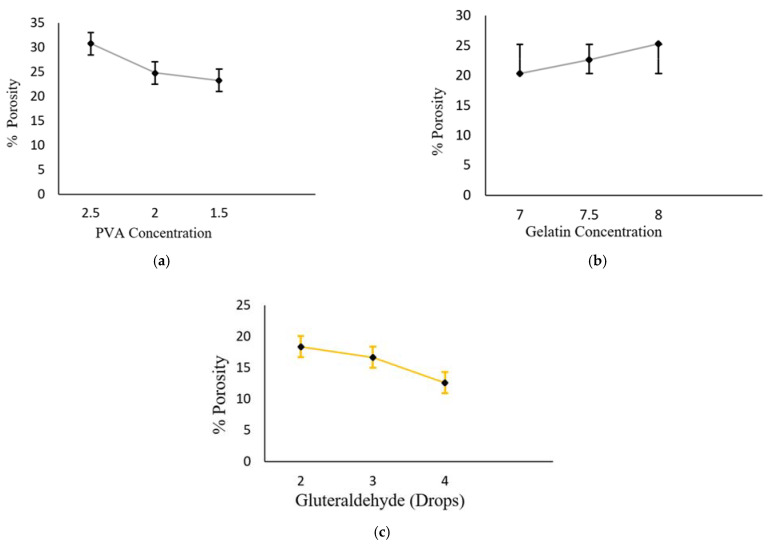
Percentage of porosity of Gel/PVA hydrogel with (**a**) decreasing concentration of PVA, (**b**) increasing concentration of Gel, (**c**) increasing quantity of glutaraldehyde. Data are expressed as mean ± S.D. and *p* < 0.01 is considered statistically significant.

When the concentration of PVA increases while keeping the concentration of Gel constant, from S1 to S3, there is a significant decrease (*p* ≤ 0.05) in the porosity of the hydrogel discs. The maximum degree of porosity was found in sample A1 (30.76 ± 1.350), a moderate degree was found in sample A2 (24.75 ± 1.327) and a minimum was found for sample A3 (23.24 ± 1.054), as shown in Figure 3a. From S4 to S6, the Gel concentration was gradually increased, while PVA was kept constant, then there occurred a significant gradual increase in the percentage of porosity of the hydrogel formulation. A6 had an increased degree of porosity, i.e., 28.27 ± 1.387, it was moderate for A5, i.e., 22.63 ± 1.108 and lower for A4, i.e., 20.36 ± 1.388, as shown in Figure 3b and when the polymers’ concentration became constant and an increasing concentration of the cross-linker (glutaraldehyde) was used, a decrease in the percentage of porosity of the hydrogel formulations from A7 to A9 was noted as 18.35 ± 1.083, 16.66 ± 1.4512 and 10.60 ± 0.450, respectively, as shown in Figure 3c.

### 3.5. In-Situ Drug Loading 

The loading capacity was determined using an in situ technique. The drug loading efficiency is an important parameter that effects a drug release profile study. The study found that the drug loading capacity of the formulations was ≈0.58 mg (A1), followed by ≈0.34 mg (A2), ≈0.50 mg (A3), ≈0.53 mg (A4), ≈0.51 mg (A5) and ≈0.52 mg (A6). The highest amount of loaded drug was obtained from the formulation (A1) that also showed the highest amount release profile in the drug release study as well. 

### 3.6. Percentage of Drug Release Profile 

**Figure 4:**
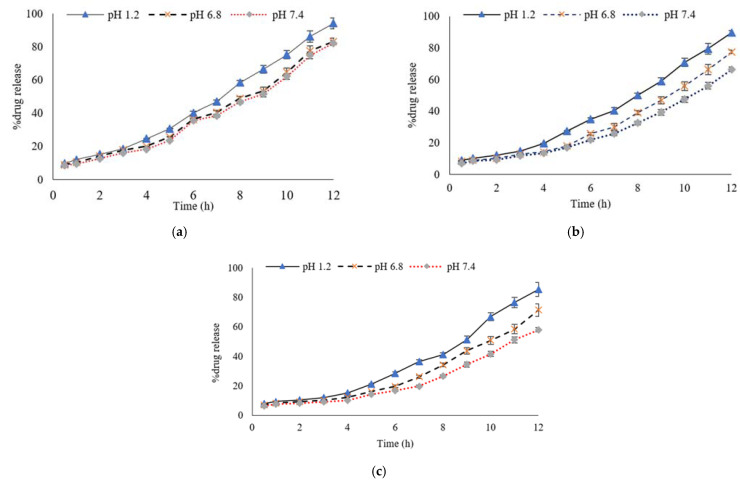
Percentage of MTX release profiles from Gel/PVA Hydrogel with polymer concentrations (**a**) GEL/PVA (9/2.5 g), (**b**) GEL/PVA (9/2 g), (**c**) GEL/PVA (9/1.5 g) at pH 1.2, pH 6.8 and pH 7.4 at 37 °C. Data are expressed as mean ± S.D. and *p* < 0.01 is considered statistically significant.

The drug release profile is an important factor due to the fact that it is directly related to the therapeutic efficacy of the active drug. The drug release profile was investigated by the amount of the drug released from the hydrogel formulation after 12 h. The impact of the pH, PVA, Gel and cross-linker was evaluated. In samples A1 to A3, the Gel concentration (9 g) was kept constant, while the PVA concentration was decreased gradually (2.5, 2 and 1.5 g, respectively) to determine the effect of the PVA concentration on MTX’s release from the Gel/PVA hydrogel at different pH values of the phosphate buffer mediums. The percent drug release profile showed that, as the concentration of the PVA decreased, the drug (MTX) release also decreased, especially at pH 7.4 and vice versa. Sample A1 showed the highest percentage of MTX release of 94.30 ± 1.24 at pH 1.2, 83.21 ± 0.19 at pH 6.8 and 82.32 ± 0.48 at pH 7.4, as shown in Figure 4a, while sample A2 showed a percentage drug (MTX) release of 89.67 ± 0.12 at pH 1.2, 77.53 ± 0.98 at pH 6.8 and 66.54 ± 0.37 at pH 7.4, as shown in Figure 4b. Sample A3 showed a percentage drug (MTX) release of 85.28 ± 0.96 at pH 1.2, 71.38 ± 0.41 at pH 6.8 and 58.03 ± 0.62 at pH 7.4, as shown in Figure 4c.

**Figure 5:**
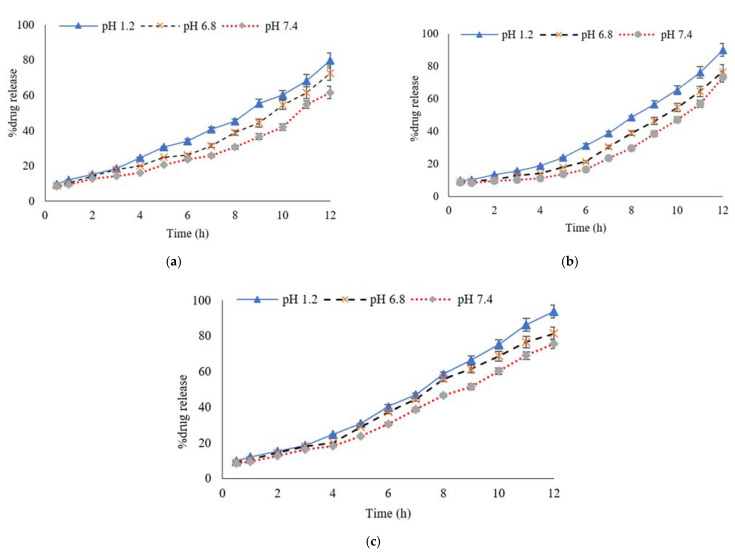
Percentage of MTX release profiles from Gel/PVA hydrogel with polymer concentrations of (**a**) GEL/PVA (7/2.5 g), (**b**) GEL/PVA (7.5/2 g), (**c**) GEL/PVA (8/2 g) at pH 1.2, pH 6.8 and pH 7.4, at 37 °C. Data are expressed as mean ± S.D. and *p* < 0.01 is considered statistically significant.

In samples A4 to A6 the PVA concentration (2 g) was kept constant, while the Gel concentration increased gradually (7, 7.5 and 8 g) to investigate the effect of the Gel concentration on MTX’s release from the Gel/PVA hydrogel formulations at different pH values of the phosphate buffer mediums. The percent drug release profile results showed that, as the concentration of the Gel increased, the drug (MTX) release also increased, especially at pH 1.2 due to the pH sensitivity of Gel. These results are also in correlation with the swelling studies. Sample A4 showed a percentage drug (MTX) release of 79.89 ± 0.56 at pH 1.2, 72.03 ± 0.12 at pH 6.8 and 61.63 ± 0.07 at pH 7.4, as shown in Figure 5a. While sample A5 showed a percentage drug (MTX) release of 89.96 ± 0.10 at pH 1.2, 76.66 ± 0.63 at pH 6.8 and 73.39 ± 0.56 at pH 7.4, as shown in Figure 5b, sample A6 showed a percentage drug (MTX) release of 93.75 ± 0.13 at pH 1.2, 81.40 ± 0.59 at pH 6.8 and 75.65 ± 0.75 at pH 7.4, as shown in Figure 5c.

### 3.7. FT-IR (Fourier Transform Infrared Spectroscopy)

**Figure 6:**
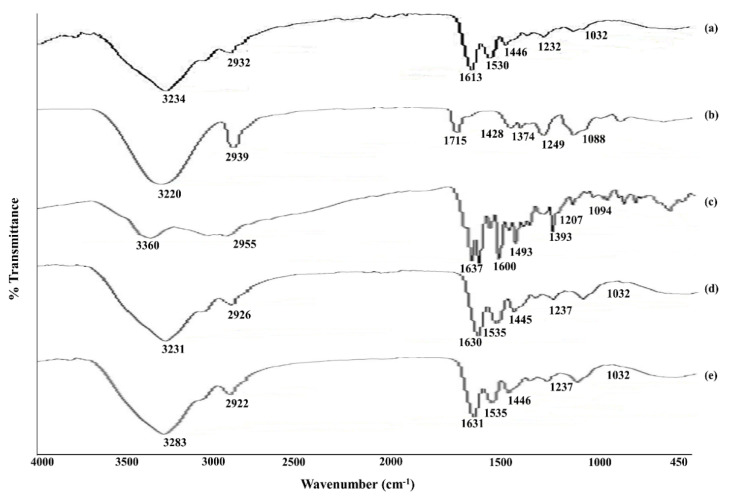
FTIR spectra of (**a**) pure PVA, (**b**) pure Gel, (**c**) MTX, (**d**) unloaded Gel/PVA hydrogel, (**e**) MTX-loaded Gel/PVA hydrogel.

FT-IR is important to detect a range of functional groups and is sensitive to changes in molecular structure. It provided information based on the chemical composition and physical state of the formulation, such as the main changes upon polymerization or cross-link. The various peaks of the Gel polymer were found to originate at 3284 cm^−1^, indicating the stretching of the -N-H group of secondary amides; at 2932 cm^−1^, indicating the C-H stretching group; at 1631.3 cm^−1^, indicating the stretching of the C=O, amide I and C-N stretching groups; at 1530 cm^−1^, indicating -N-H bending and the peak at 1237.6 cm^−1^ indicates the amide-III [44], as shown in Figure 6a. The FTIR spectra of the pure PVA showed a peak of a broad band at 3320.74 cm^−1^, which may belong to the O-H stretching and might be due to inter and intra molecular hydrogen bonds. A peak at 2939.89 cm^−1^ shows the vibrational bend due to the stretching of the C-H bond from the alkyl groups. The peak at 1715.73 cm^−1^ indicates C=O due to the strong carboxylic group. The peaks at 1485 cm^−1^ and 1342 cm^−1^ indicate the scissoring -CH2 group and vibrational bending -OH group [28], respectively, as shown in Figure 6b. The peak at 3360.70 cm^−1^ indicates the stretching of the O-H from the carboxyl group, the peak at 2955.9 cm^−1^ indicates the N-H stretching of the primary amine, the peaks from 1670 cm^−1^ to 1600 cm^−1^ indicate the -C=O stretching of the amide group and the carboxylic group, the peak at 1493.63 cm^−1^ indicates the -N-H bending from the amide group, the peaks from 1400 cm^−1^ to 1200 cm^−1^ indicate the stretching of -C-O from the carboxylic group [45], as shown in Figure 6c. As the functional chemical groups of the Gel/PVA occur in the same peak regions, they are with overlapping over one another and, therefore, there no significant chemical interactions occurred, as shown in Figure 6d. The MTX drug was water insoluble and showed no chemical interaction with the hydrogel, which is evident from the FTIR spectra of the MTX-loaded Gel/PVA hydrogel sample, as shown in Figure 6e.

### 3.8. Thermal Gravimetric Analysis (TGA)

**Figure 7:**
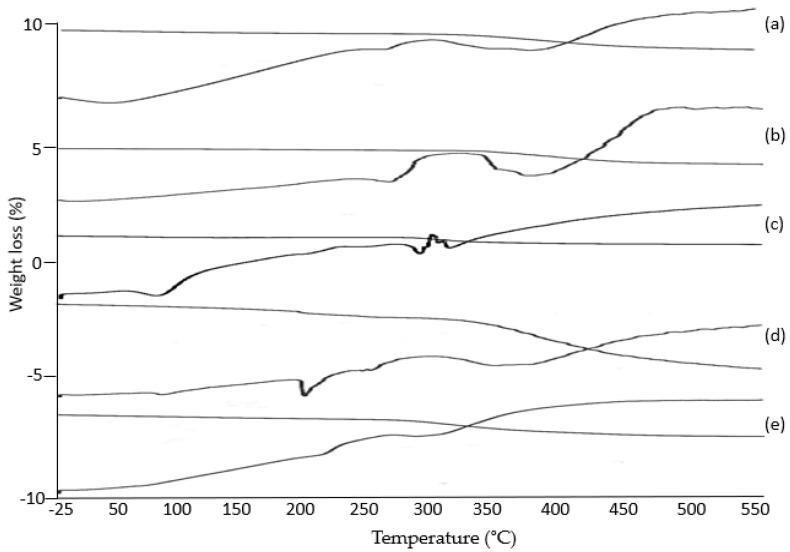
TGA spectra of (**a**) pure Gel, (**b**) pure PVA, (**c**) MTX, (**d**) unloaded Gel/PVA hydrogel, (**e**) MTX-loaded Gel/PVA hydrogel.

TGA was performed to obtain data for any changes in the physical and chemical properties of formulations such as the thermal or oxidative stability, the decomposition profile, moisture and the loss of volatiles of the content. An endothermic peak at 180 °C indicates the melting point of the amino acid. The broad endotherm at 250 °C indicates the decomposition of the protein. TGA shows an approximately 15% weight loss up to 150 °C due to the loss of water. A steep change in the weight of the sample over a temperature range of 250 °C to 350 °C, characterized by 35% weight loss, is related to the thermal decomposition of the protein, as shown in Figure 7a. The thermogram of PVA shows an endothermic event at 180 °C, which is related to the glass transition of the polymer. Another endotherm at 250 °C, characterized by the 30% weight loss in the TGA, is linked to the decomposition of the samples, as shown in Figure 7b. The thermogram of MTX demonstrates an endothermic peak at 100 °C, which is ascribed to the dehydration of the sample. The second endothermic peak at 170 °Cis due to the melting of the drug substance. A noisy endotherm at 240 °C is possibly linked to the thermal decomposition of the sample. TGA data are also correlated to the weight loss at 100 °C due to dehydration and thermal degradation at 240 °C, as shown in Figure 7c. The DSC thermogram indicates an endothermic peak at 80 °C, which is the dehydration of the sample. The sharp endothermic peak at 170 °C indicates the melting of the formulation. The broad endotherm from 250 to 350 °C shows the thermal decomposition of the sample. The TGA results indicate a low gradient of weight change at up to 150 °C due to the removal of water. A small yet abrupt change in the sample weight at 170 °C matches the enthalpic shift due to the melting of the sample. Likewise, a 50% reduction in the sample weight at a temperature range of over 250 °C and onward refers to the thermal decomposition of the mass, as shown in Figure 7d. The DSC profile shows an endothermic peak at 220 °C due to fusion, while the second endotherm at 260 °C is due to the decomposition of the sample. The TGA profile compliments the DSC and small changes in weight are evident up to 200 °C. Thermal decomposition is recorded at temperatures exceeding 200 °C, reflecting the thermal stability of the sample, as shown in Figure 7e.

## 4. Discussion

Gelatin is one of the most studied biomaterials as it is well characterized, biodegradable and water-soluble [46]. The pka values of the acidic components of the polymers and the pH of the buffer medium used for the swelling of the hydrogel might play a significant role in the determination of the swelling behavior of the hydrogel. The swelling properties of the hydrogel’s polymer network are greatly affected by the pH of the surrounding buffer medium. In a swelling kinetic study of the Gel/PVA hydrogel, the primary amino groups of the polymers are ionized at a lower pH (acidic pH), below the pka value of the polymers. Then, swelling might have occurred due to the protonation of the polymer’s amino groups. After the protonation of the –NH_2_ groups, positively charged –NH_3_^+^ groups evenly distributed in a network of hydrogel polymers. When the concentration of these positively charged groups increases, a difference is created in the osmotic pressure of the inner and outer environment of the hydrogel’s polymer network. These positively charged groups then started repulsion among the polymer’s chains of the gel network, which could lead to the hydrogel network swelling more [47]. The degree of swelling, both dynamic and equilibrium, was observed to increase in an acidic medium with pH 1.2, which may be due to the –NH_2_ groups of the Gel polymer in the Gel/PVA hydrogel and the basic nature of the amino groups that are ionized (protonated) at a pH lower than the pka value of Gel. It can result in the production of positively charged groups of –NH_3_^+^ that repel each other and may result in an increased swelling of the hydrogel at a lower pH [48]. In a medium with a pH value higher than an acidic pH value, an increase in the swelling of the hydrogel network occurs.

At the basic pH of the dissolution medium, the increasing equilibrium of the Gel/PVA hydrogel may be due to the –OH groups of the polymer in the gel network [49]. With an increase in the PVA concentration, there is an increase in the swelling of the hydrogel. The functional groups of PVA, i.e., the –OH group, may actually be responsible for the swelling of the hydrogel because these functional groups are hydrophilic in nature [50]. PVA is a water-soluble polymer; therefore, when its concentration increases, equilibrium swelling occurs very early, because when the PVA quantity increases its chains are highly hydrated due to their hydrophilic nature [51]. During the swelling kinetics study of the Gel/PVA hydrogel, it was observed that both the dynamic and equilibrium swelling were decreased in both the acidic and basic pH media, as the concentration of the glutaraldehyde increases due to the number of cross-linking polymer chains. When the concentration of glutaraldehyde increases in the gel formulation, the –OH groups of PVA and more –NH_2_ groups of the Gel are consumed in cross-linking reactions, and the aldehyde group of the cross-linker reacts with the –OH group of PVA to form an acetal group and the –NH_2_ group of Gel for a Schiff base. After these cross-linking reactions, the PVA hydroxyl groups tend to form hydrogen bonding with water molecules and, thus, result in the decreased swelling of the hydrogel. These findings can be correlated with the results found previously in a correlation study of Gel/PVA [52]. When the PVA concentration increased in the formulation as compared to Gel, more swelling was observed [53]. 

For the determination of the non-crossed linked polymer fraction of the Gel/PVA hydrogel formulation, the percentage sol-gel fraction analysis method was adopted. After taking the results of all the samples, it was observed that, by increasing the concentration of PVA, the percentage gel fraction was increased and vice versa [54]. By increasing the polymer Gel concentrations, an increase in the percentage gel fraction and a decrease in the sol fraction can occur [55]. Adding or increasing the concentration of glutaraldehyde in the formulations also resulted in an increase in the percentage gel fraction, because the cross-linker caused an increased entanglement of the polymer chains of the gel formulation [56]. Entering the solvent of the buffer medium into the pre-existing pores or the spaces formed thermodynamically between the chains of the polymers of the gel formulation is the diffusion coefficient. Therefore, the diffusion coefficient may also be directly proportional to polymer’s concentration. Increasing the polymer concentration of either PVA or Gel in the hydrogel formulations may result in an increase in the diffusion coefficient and vice versa. While the diffusion coefficient is inversely proportional to the glutaraldehyde quantity [57], the porosity of the Gel/PVA hydrogel increases when the concentration of the Gel/PVA polymer increases. This can be attributed to the increase in the polymer concentration that increases the viscosity of the gel solution. During the preparation of that solution, bubbles also formed in the solution and, due to its viscous nature, the bubbles were entrapped in the formulation, which acts as an interconnected pore channel for the hydrogel that results in an increase in the porosity of the gel. Increasing the glutaraldehyde cross-linker concentration will lead to a decrease in the spaces among the polymer chains by cross-linking them, which, thus, results in a decrease in the spaces and pores and, thus, the percentage porosity of the gel [58].

The MTX drug release from the Gel/PVA hydrogel formulations followed the swelling controlled release mechanism [27,59]. During swelling, the hydrogel discs might swell up and can change shape with time in the buffer solution. The shape transition of the hydrogel discs also depends on the pH of the swelling medium, the diffusion coefficient, the quantity of polymers (Gel/PVA) and the porosity. The solvent of the buffer medium may enter the hydrogel disc and, thus, the disc swells up and releases the drug [60]. The FTIR spectra of the drug-unloaded hydrogel may show no definite change (significant chemical interactions) from the Gel/PVA spectra. This might be due to the fact that the concentration of the PVA polymer is lower; therefore, it will not affect the drug-unloaded hydrogel’s FTIR spectra [61,62] or most of the functional chemical groups occurring in the same peak regions, as they have no cross-linker in the drug-loaded samples (A1-A6) and, therefore, they may only overlap with each other [63]. While the FTIR spectra of the drug-loaded hydrogel (Figure 3) may also show no significant variation from the PVA spectra, because MTX is water insoluble, it may not interact with the water-soluble polymers (either Gel or PVA) [64].

## 5. Conclusions

Hydrogels of different Gel/PVA polymer and cross-linker (glutaraldehyde) concentrations were observed for their swelling profile, porosity, sol-gel fraction, diffusion coefficient and percent drug release. In this study, the swelling behavior of the hydrogels reflected their pH sensitivity and the effect of the polymer to cross-linker ratio as well. The release of MTX from the gels was sustained and in a controlled release manner relating to its pH sensitivity. The Gel/PVA hydrogel formulations exhibited a high pH sensitive, characteristic of time dependent drug release that may have practical applications in delivering MTX to a colon site for colorectal cancer treatment. This study found several major points that can be considered unique to this hydrogel formulation, such as drug release rate, cross-linking degree and loading efficiency. Furthermore, these hydrogels can be the ideal cargo for other active pharmaceutical ingredients that need to be released at the colon site. Despite huge improvements, a few parameters such as absorption, plasma half-life and bioavailability could be studied for further investigation to develop a novel oral delivery system.

## Data Availability

Data will be available upon request.
